# A genome-wide survey of Major Histocompatibility Complex (MHC) genes and their paralogues in zebrafish

**DOI:** 10.1186/1471-2164-6-152

**Published:** 2005-11-04

**Authors:** Jennifer G Sambrook, Felipe Figueroa, Stephan Beck

**Affiliations:** 1 Wellcome Trust Sanger Institute, Genome Campus, Hinxton, Cambridge CB10 ISA, UK; 2 Max-Planck-Institut für Biologie, Abteilung Immunogenetik, 72076 Tübingen, Germany

## Abstract

**Background:**

The genomic organisation of the Major Histocompatibility Complex (MHC) varies greatly between different vertebrates. In mammals, the classical MHC consists of a large number of linked genes (e.g. greater than 200 in humans) with predominantly immune function. In some birds, it consists of only a small number of linked MHC core genes (e.g. smaller than 20 in chickens) forming a minimal essential MHC and, in fish, the MHC consists of a so far unknown number of genes including non-linked MHC core genes. Here we report a survey of MHC genes and their paralogues in the zebrafish genome.

**Results:**

Using sequence similarity searches against the zebrafish draft genome assembly (Zv4, September 2004), 149 putative MHC gene loci and their paralogues have been identified. Of these, 41 map to chromosome 19 while the remaining loci are spread across essentially all chromosomes. Despite the fragmentation, a set of MHC core genes involved in peptide transport, loading and presentation are still found in a single linkage group.

**Conclusion:**

The results extend the linkage information of MHC core genes on zebrafish chromosome 19 and show the distribution of the remaining MHC genes and their paralogues to be genome-wide. Although based on a draft genome assembly, this survey demonstrates an essentially fragmented MHC in zebrafish.

## Background

The human Major Histocompatibility Complex (MHC) is a gene-dense region on chromosome 6p21.3, and comprises a group of genes that are involved functionally with the adaptive and innate immune system. From centromere to telomere, it is divided into five regions: extended class II, classical class II, class III, classical class I and extended class I. The classical MHC contains 224 genes, many of which are pseudogenes, and with every two in five expressed genes having a potential immune function and a role in disease resistance [1], this region has become a focal locus in comparative genomics. Antibody and T cell mediated immune responses against invading pathogens are initiated through MHC class I and class II molecules [2]. These main components are not only missing from invertebrates, but are also not present in primitive jawless fish, such as hagfish and lamprey [3]. MHC class I and II molecules do, however, exist in all jawed vertebrates, including the cartilaginous fish. The gene loci in the class III region encode a variety of proteins with both immune and non-immune functions [4].

Genomic sequences encompassing the MHC are currently available for human, chimpanzee, macaque, rat, mouse, cat, pig, horse, quail, chicken, frog, teleost fish and shark [5]. While the genomic architecture of the mammalian MHC is conserved, the number of genes between species can differ greatly. In birds, for example, the minimal essential MHC in chicken consists of 19 genes [6] while gene duplications have expanded this region in quail [7] and sparrows [8]. The linkage of class I, II, and III region genes can be traced back to cartilaginous fish, which are the earliest jawed vertebrates known to have diverged from a common ancestor with humans [9]. MHC loci, however, do not always exist in a single tightly linked cluster as generally observed in mammals. A large scale inversion has separated the class IIB cluster from the MHC-linked class IIA cluster in cattle [10]. The class I and class II loci in zebrafish (*Brachydanio rerio*) are found on different chromosomes [11,12]. A similar organisation is present in trout, stickleback, common guppy and cichlid fish [13,14]. This observation demonstrates that the separation of the MHC class I and class II loci is characteristic of teleost fish, which represent half of all vertebrates. Since the genes of the immune system were present in the common ancestor of tetrapods and teleosts, the differences in their genomic organisation may be the result of lineage-specific chromosomal events such as duplications, inversions, deletions and translocations.

The genome of the zebrafish is currently being sequenced at the Wellcome Trust Sanger Institute. The availability of sequence data will allow an insight into the understanding and evolution of the immune system in fish. To date, small regions containing major histocompatibility genes have been mapped by radiation hybrid mapping and sequencing of select genomic clones [15,16]. With the ongoing whole-genome sequencing efforts and the compilation of physical maps, it is now possible to examine larger genomic regions and assess the degree of shared synteny between mammals and fish on a genome-wide scale. Here we have utilised a comprehensive whole-genome shotgun assembly data set to fully analyse the zebrafish loci that are related to the human MHC. The human genome contains at least three regions that are paralogous to the MHC [17]. These are thought to be the result of two rounds of duplications that occurred early during vertebrate evolution. In this study, we examine the genome-wide distribution of paralogous genes in zebrafish.

## Results and Discussion

### Analysis of the MHC class I region in zebrafish

The MHC class I region in human embodies gene clusters coding for HLA class I molecules, histones, solute carriers, vomeronasal receptors, olfactory receptors and zinc fingers [18]. These clusters have undergone a large-scale expansion and each has paralogues throughout the genome. Ten representative members of the extended class I region, which are expressed and have paralogues in the human genome, and more than 30 genes that reside in the classical class I region were chosen for this analysis (Table 1). Genes that were excluded from this study included pseudogenes, zinc fingers with the tripartite motif (TRIM), and RING finger proteins. Approximately half of the human genes were identified in the zebrafish whole-genome assembly, predominantly on chromosome 19. Because multigene families evolve by a birth-and-death process [19], orthologous genes are usually difficult to identify through sequence similarity searches.

**Table 1:** List of human MHC class I and extended class I (xI) region genes used against the zebrafish whole-genome assembly. Genes with the suffix "like" are either gene fragments and/or highly similar to their human counterparts. Genes not identified by sequence similarity searches are marked as not found (NF). The official gene nomenclature for zebrafish is shown in brackets.

MHC CLASS I REGION GENES						
HUMAN			ZEBRAFISH			
	
	name	accession	location	location	location	location
xI	HFE	NM_000410	NF			
xI	HMGN4	NM_006353	NF			
xI	PRSS16	NM_005865	NF			
xI	POM121L2	NM_033482	NF			
xI	GPX5	NM_001509	NF			
xI	RFP	NM_006510	NF			
xI	MAS1L	NM_052967	NF			
xI	UBD	NM_006398	NF			
xI	GABBR1	NM_021905	15 [gabbr1]	19 [gabbr1]		
xI	MOG	NM_002433	NF			
I	HLA*		19 [mhc1uea]	19 [mhc1uaa]	19 [mhc1ufa]	19 [mhc1uda]
			s_1723 [mhc1ze]	1 [mhc1-like] (X3)	1 [zgc:64115]	
I	HCG9	NM_005844	NF			
I	PPP1R11	NM_021959	NF			
I	RPP21	NM_024839	NF			
I	GNL1	NM_005275	16 [gnl1]			
I	PRR3	NM_025263	NF			
I	ABCF1	NM_001090	19 [zgc:85667]			
I	PPP1R10	NM_002714	19 [ppp1r10]			
I	MRPS18B	NM_014046	19 [mrps18b]			
I	C6orf134	NM_024909	19 [c6orf134]			
I	C6orf136	NM_145029	19 [c6orf136]			
I	DHX16	NM_003587	NA5816 [dhx16]	15 [dhx16]		
I	NRM	NM_007243	NA1826 [nrm]			
I	MDC1	NM_014641	NF			
I	TUBB	NM_178014	20 [tubb5]	14 [tubb2]	23 [tubb2]	
I	FLOT1	NM_005803	19 [flot1]	16 [flot1b]		
I	IER3	NM_003897	NF	22 [ier5]		
I	DDR1	NM_013994	16 [ddr1-like]	4 [ddr2]		
I	GTF2H4	NM_001517	19 [zgc:77721]			
I	VARS2L	NM_020442	19 [vars2-like]			
I	DPCR1	NM_080870	NF			
I	C6orf15	NM_014070	NF			
I	CDSN	NM_001264	NF			
I	PSORS1C1	NM_014068	NF			
I	PSORS1C2	NM_014069	NF			
I	C6orf18	NM_019052	NF			
I	TCF19	NM_007109	1 [tcf19-like]			
I	POU5FI	NM_002701	16 [pou5f1]			
I	MICA	NM_000247	NF			
I	HCP5	NM_006674	NF			
I	MICB	NM_005931	NF			

**Figure 1:**
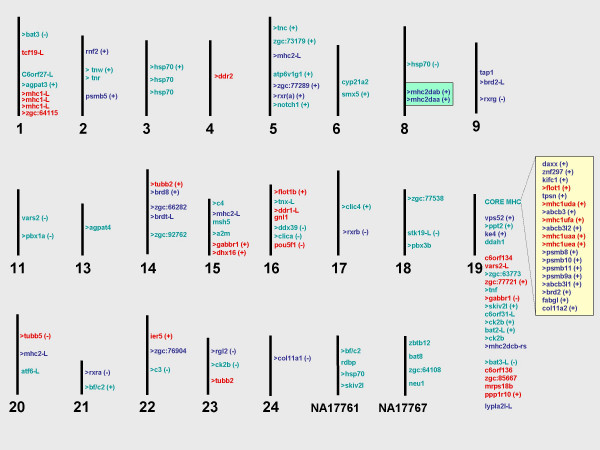
Map of major histocompatibility genes and their paralogues in zebrafish (not to scale). Only chromosomes and two unmapped contigs (NA17761 and NA17767) that harbour MHC-related genes are shown. Orthologues or paralogues of human MHC class I region genes are shown in red, of class II region genes in blue, and class III region genes in green. Genes with the suffix "L" for like are either gene fragments and/or highly similar to their human counterparts. Genes that have more than one copy in the genome are shown by a greater than (>) symbol. Similarities and differences between the whole-genome assembly and mapping information present in the ZFIN database are shown by plus (+) and minus (-) signs, respectively. The core MHC has been compiled from five genomic clones spanning this region [EMBL:AL672216, EMBL:AL672151, EMBL:AL672164, EMBL:AL672176].

Chromosome 19 harbours the largest number of MHC genes in zebrafish (Figure 1). The core region, comprising genes encoding the classical peptide presenting class I molecules (*mhc1uea*, *mhc1uaa*, *mhc1ufa*, *mhc1uda*), proteasome subunits (*psmb8, psmb9a*, *psmb11*, *psmb10*), ATP-binding cassettes (*abcb3, abcb3l1*, *abcb3l2*) and tapasin (*tpsn*), cluster tightly together and span approximately 175 kb of DNA. Over 5 Mb downstream of these loci are several other genes that span the human MHC region, although these are not all found adjacent to each other. In humans, MHC class I molecules are expressed on the surface of most cells and are recognized by CD8+ cytotoxic T cells. They are heterodimers composed of an alpha subunit, encoded by the MHC, and a beta subunit, or b-2 microglobulin (*B2M*), which maps to chromosome 15q21–q22.2. In zebrafish too, the *b2m *gene is found located in a different part of the genome on chromosome 13. Previously unmapped class I ZE lineage genes [20] and novel MHC class I antigen fragments have been identified on chromosome 1 and scaffold 1723. The additional functional class I lineage (*mhc1ze*) is also present in two other cyprinids (carp and barbus), and not linked to other class I and II genes.

### Analysis of the MHC class II region in zebrafish

MHC class II molecules are heterodimers comprising A (alpha) and B (beta) chains that present peptides to CD4+ T cells via the endosomal pathway. One class IIA (*mhc2daa*) and six class IIB (*mhc2dab*, *mhc2dbb*, *mhc2dcb*, *mhc2ddb*, *mhc2deb*, *mhc2dfb*) genes have previously been identified by screening a zebrafish genomic BAC library [21]. Only the *mhc2dab *and *mhc2daa *genes are known to be expressed [22]. They are closely linked and were identified on chromosome 8 (Table 2). Analysis of approximately 1 Mb of contiguous DNA surrounding the functional class II region in zebrafish demonstrates the presence of 23 flanking genes mapping to various human chromosomal locations, including two or more genes mapping to human 6q (*C6orf117*, *LOC557721*, *ppil4*), 12q (*slc15a4*, *KIAA1944*), 20q (*rnpc2*, *slc12a5*), 22q (*slc7a4*, *sf3a1*) and Xp (*pqbp1l*, *t541*, *jarid1c*). The lack of genes mapping to the human MHC, in addition to the low gene density of this region, indicates that the functional zebrafish class II region is the result of a translocation event [12].

**Table 2:** List of human MHC class II and extended class II (xII) region genes used against the zebrafish whole-genome assembly. Genes with the suffix "like" are either gene fragments and/or highly similar to their human counterparts. Genes not identified by sequence similarity searches are marked as not found (NF). The official gene nomenclature for zebrafish is shown in brackets.

MHC CLASS II REGION GENES						
HUMAN			ZEBRAFISH			
	
	name	accession	location	location	location	location
II	C6orf10	NM_006781	NF			
II	BTNL2	NM_019602	NF			
II	HLA-D*		8 [mhc2dab]	8 [mhc2daa]	19 [mhc2dcb-rs]	s_2399 [mhc2dfb]
			5 [mhc2-like]	NA14232 [mhc2dbb]	NA6696 [mhc2ddb]	NA6696 [mhc2-like]
			20 [mhc2-like]	15 [mhc2-like]		
II	TAP2	NM_018833	19 [abcb3]	19 [abcb3l1]	19 [abcb3l2]	
II	PSMB8	NM_004159	19 [psmb8]	19 [psmb10]	19 [psmb11]	19 [psmb9a]
			2 [psmb5]	s_1793 [psmb7]		
II	TAP1	NM_000593	9 [tap1]			
II	PSMB9	NM_002800	19 [psmb8]	19 [psmb10]	19 [psmb11]	19 [psmb9a]
			2 [psmb5]	s_1793 [psmb7]		
II	BRD2	NM_005104	19 [brd2]	9 [brd2-like]	s_549 [brd3]	5 [zgc:77289]
			14 [brd8]	14 [brdt-like]	s_277 [brd4]	
xII	COL11A2	NM_080679	19 [col11a2]	24 [col11a1]		
xII	RXRB	NM_021976	17 [rxrb]	NA16779 [rxrb]	21 [rxra]	5 [rxra]
			9 [rxrg]			
xII	SLC39A7	NM_006979	19 [ke4]			
xII	HSD17B8	NM_014234	19 [fabgl]			
xII	RING1	NM_002931	NF	2 [rnf2]		
xII	VPS52	NM_022553	19 [vps52]			
xII	RPS18	NM_022551	NA303 [rps18]			
xII	B3GALT4	NM_003782	NF	22 [zgc:76904]	s_1722 [b3galt1]	
xII	C6orf11	NM_005452	s_2182 [bing4]			
xII	HKE2	NM_014260	14 [zgc:66282]	NA8827 [zgc:66282]		
xII	RGL2	NM_004761	23 [rgl2]	NA14862 [rgl2-like]	NA7773 [zgc:77299]	NA12682 [ralgds]
xII	TAPBP	NM_003190	19 [tpsn]			
xII	ZNF297	NM_005453	19 [znf297]			
xII	DAXX	NM_001350	19 [daxx]			
xII	LYPLA2L	CAB63783	19 [lypla2l-like]			
xII	KIFC1	NM_002263	19 [kifc1]			

Mapping to chromosome 19, approximately 22 Mb telomeric of the class I region, is the class IIB gene *mhc2dcb-rs *(Q95HJ7), with a similar sequence on contig NA4006. Also within this segment of DNA there are two predicted class IIA chain-encoding gene fragments, consisting of only the alpha2 domain and cytoplasmic tail-encoding parts. It is therefore unlikely that this locus is functional. Assuming that these gene fragments are not due to errors in the whole-genome assembly, it is evidence for a linkage of remnants of MHC-related class II genes with the core MHC region containing the class I peptide presenting genes.

Previously identified *mhc2dbb *and *mhc2dfb *genes are predicted to be located in currently unmapped contigs, NA14232 and scaffold 2399. However, *mhc2deb *(found in clone U08874) was not identified in the current assembly, and may be attributed to gaps in the sequence data or allelic or haplotypic differences. Several fragmented sequences resembling class IIA and/or B chain-encoding genes were also identified in contigs NA17244 and NA15003, and chromosomes 5, 15 and 20. Only contig NA6696 harbours putative complete class IIA and class IIB genes. Another gene found in this 15 kb contig resembles *mhc2ddb*, consisting of exons 3 and 4 only.

The two TAP transporters in human, namely *TAP1 *and *TAP2*, are both located in the MHC class II region, and are closely linked to genes encoding the proteasome subunits and the class II molecules. In zebrafish, however, a *TAP1-like *sequence is found on chromosomes 9 and three *TAP2*-*like *genes are found in the class I region [15] on chromosome 19. The latter, named *abcb3*, *abcb3l1*, *abcb3l2*, form part of the zebrafish core MHC region. The trout *tap1 *gene is not linked to the major class IA region either [23]. Likewise, in Fugu, the *tap1 *gene is found on an isolated scaffold that is not linked to the main class I region [24].

### Analysis of the MHC class III region in zebrafish

The human class III is the most gene dense region within the MHC containing few or no pseudogenes. Unlike the class I and class II loci that are evolutionary and functionally related [25], the class III region genes are not. The class III, however, include immune-related genes such as those encoding complement components, tumour necrosis factors and heat shock proteins. The search for the MHC class III region in zebrafish was first initiated by the identification of the *BF/C2 *gene using degenerate PCR [26]. This was then followed by the identification of several, but not all, of the zebrafish homologues of the human MHC class III region genes [16].

In the zebrafish, the MHC class III genes are found distributed throughout their genome (Table 3). Several zebrafish genes (*zgc:63773*, *tnf*, *bat2-like*, *ck2b*, *ddah1*, *skiv2l*, *C6orf31-like*, *ppt2, bat3-like*) map to chromosome 19, which also encompasses the largest stretch of MHC-related genes. Two other unmapped scaffolds also harbour several class III genes: *neu1*, *zgc:64108*, *bat8*, *zbtb12 *are found in NA17767; and *bf/c2*, *rdbp*, *hsp70*, *skiv2l *are in scaffold NA17761. These are syntenic to the human MHC class III region with the conservation of both gene order and content (Figure 1).

**Table 3:** List of human MHC class III region genes used against the zebrafish whole-genome assembly. Genes with the suffix "like" are either gene fragments and/or highly similar to their human counterparts. Genes not identified by sequence similarity searches are marked as not found (NF). The official gene nomenclature for zebrafish is shown in brackets.

MHC CLASS III REGION GENES					
HUMAN		ZEBRAFISH			
	
name	accession	location	location	location	location
BAT1	NM_004640	19 [zgc:63773]	16 [ddx39]		
ATP6V1G2	NM_130463	NF	5 [atp6v1g1]		
NFKBIL1	NM_005007	NF			
TNF	NM_000594	19 [tnf]	NA11949 [tnfa-like]		
LST1	NM_007161	NF			
NCR3	NM_147130	NF			
AIF1	NM_001623	NF	5 [zgc:73179]		
BAT2	NM_004638	19 [bat2-like]			
BAT3	NM_004639	1 [bat3]	19 [bat3-like]		
APOM	NM_019101	NF			
C6orf47	NM_021184	NF			
BAT4	NM_033177	s_339 [bat4-like]			
CSNK2B	NM_001320	19 [ck2b] (X2)	23 [ck2b]		
LY6G5B	NM_021221	NF			
LY6G5C	NM_025262	NF			
BAT5	NM_021160	NA9087 [bat5-like]			
LY6G6D	NM_021246	NF			
LY6G6E	NM_024123	NF			
LY6G6C	NM_025261	NF			
C6orf25	NM_025260	NF			
DDAH2	NM_013974	NF	19 [ddah1]		
CLIC1	NM_001288	18 [zgc:77538]	14 [zgc:92762]	16 [clica]	17 [clic4]
MSH5	NM_002441	15 [msh5]			
C6orf26	NM_025259	NF			
C6orf27	NM_025258	1 [c6orf27-like]			
VARS2	NM_006295	11 [vars2]			
LSM2	NM_021177	6 [smx5]			
HSPA1A	NM_005345	NA17761 [hsp70]	3 [hsp70] (X3)	8 [hsp70]	
C6orf48	NM_016947	NF			
NEU1	NM_000434	NA17767 [neu1]			
C6orf29	NM_032794	NA17767 [zgc:64108]			
BAT8	NM_025256	NA17767 [bat8]			
ZBTB12	NM_181842	NA17767 [zbtb12]			
BF/C2	NM_001710	21 [bf]	NA17761 [bf]		
RDBP	NM_002904	NA17761 [rdbp]			
SKIV2L	NM_006929	19 [skiv2l]	NA17761 [skiv2l]		
DOM3Z	NM_032419	NA17766 [dom3z]			
STK19	NM_004197	18 [stk19-like]			
C4B	NM_000592	15 [c4]	15 [a2m]	22 [c3]	NA17328 [a2m]
		NA14479 [c3]			
CYP21A2	NM_000500	6 [cyp21a2]			
TNXB	NM_032470	16 [tnx-like]	2 [tnw]	2 [tnr]	5 [tnc]
CREBL1	NM_004381	NF	20 [atf6-like]		
FKBPL	NM_022110	NF			
C6orf31	NM_030651	19 [c6orf31-like]			
PPT2	NM_005155	19 [ppt2]	s_1796 [ppt2]	NA5816 [zgc:55621]	
EGFL8	NM_030652	NF			
AGPAT1	NM_032741	NF	1 [agpat3]	13 [agpat4]	
RNF5	NM_006913	NF			
AGER	NM_001136	NF			
PBX2	NM_002586	NA14559 [pbx1a]	11 [pbx1a]	18 [pbx3b]	NA11844 [pbxy]
GPSM3	NM_022107	NF			
NOTCH4	NM_004557	s_285 [notch3]	5 [notch1]	NA15389 [notch2]	s_1523 [notch3]

Approximately half of the human MHC class III region genes were not identified in the zebrafish assembly. Among these were the *Ly6 *family members, which may therefore be mammalian-specific. Alternatively, being involved in the immune response, *Ly6 *genes evolved more rapidly than others, and might have diverged sufficiently to not be recognised by sequence similarity searches. For eight human genes, *ATP6V1G2*, *AIF1*, *CLIC1*, *CREBL1, DDAH2, AGPAT1*, *PBX2 *and *NOTCH4*, the paralogues but not the orthologues of the genes in the human MHC class III region have been identified by sequence similarity in zebrafish. This observation extends to *IER3, RING1 *and *B3GALT4 *found in the class I and extended class II regions. The presence or absence of other genes may be attributed to lineage-specific evolution. For example, lamprey [27], zebrafish [26] and medaka [28] possess genes equally similar to both mammalian *BF *and *C2*, while a *Xenopus *clone has clearly been identified as encompassing *BF*. This *BF/C2 *ancestral gene has further duplicated in zebrafish [29], copies of which were identified on chromosome 21 and contig NA17761. Similarly, a recent survey of *hsp70 *genes in *Xiphophorus maculates *(platyfish) [30] has revealed that a single *HSP70 *gene gave rise to four distinct groups of genes: mammalian testis-specific *HST70*, mammalian MHC-linked *HSP70*, mammalian *HSP70B' *and the fish *HSP70*. Human class III *HSPA1A*, *HSPA1B *and *HSPA1L *genes are intronless and encode identical or near-identical proteins. Intronless zebrafish *hsp70 *genes were identified on chromosome 8 and scaffold NA17761. Three additional copies of *HSP70*-*like *genes were identified on chromosome 3, although these contained one, two or three introns in the 3' end of their sequence. The identified zebrafish genes cluster phylogenetically within the fish subgroup, apart from the sequence on scaffold NA17761, which appears to be similar to the mammalian MHC-linked heat shock protein sequences (data not shown).

### Construction of in-silico gene maps

The zebrafish MHC gene map (Figure 1) was constructed using primarily mapping information from the whole-genome assembly displayed in the Ensembl platform. The position of at least 49 genes mapped in this survey could be verified by comparison to experimental data stored in the ZFIN database, which also yielded information for genes residing in unmapped contigs. Mapping data from these two sources did not coincide for several genes, including *bat3*, *clica*, *vars2*, *c3*, *pou5f1*, *rgl2*, *tubb5*, *ddx39*, *col11a1*, *pbx1a*, and *stk19-like*. These discrepencies may be attributed to the high levels of polymorphisms and regions of misassembly caused by the source DNA used for the whole-genome assembly being collected from over 1000 embryos. Until the genome sequence is complete, it will not be possible to accurately predict the position and number of all human MHC orthologues in zebrafish. There are also a number of genes, notably *CD1*, *MOG *and *HFE*, that could not be identified in the draft assembly used here (these are listed as 'not found' in Tables 1, 2, 3).

There were a number of difficulties associated with assessing the degree of synteny between the zebrafish whole-genome assembly and the human MHC. Although BLAST is a heavily used analysis tool for identifying related sequences, it does not discriminate between large gene family members. To maximise the identification of MHC-related loci in zebrafish, only the highest BLAST scoring sequences were chosen for further analysis (many of which were unique BLAST reciprocal hits), and searches were conducted using blastp or tblastx. Orthology can be confirmed by obtaining mapping data of surrounding genes, with the assumption that groups of syntenic genes would remain in close proximity through evolution, and therefore be maintained in similar segments of DNA. The physical linkage between numerous MHC-related loci is apparent on zebrafish chromosome 19. Genes mapping to zebrafish chromosome 5 have also been observed to be syntenic with human chromosome 9 [31]. Gene blocks, in particular present in contig NA17761 and NA17767, map closely together in the human MHC class III region. This criterion is more difficult to apply when genes are dispersed, as seen in Figure 1. The Ensembl platform [32] used to map the MHC in zebrafish provides a combination of alignment data, genomic location, detailed transcript structures to compare functional domains of orthologous proteins, in conjunction with multi-species comparisons. In combination with further phylogenetic analyses when duplicates of one gene were identified, the distinction between orthologues and paralogues was ascertained. Nevertheless, it is very difficult to assign orthologous comparative relationships with multigene families until all members of the family have been sequenced in both organisms. This might be the case in human, but the zebrafish genome is yet to be completed.

### Duplicated genes and MHC paralagous regions

In human, it was observed that four regions, the MHC on 6p21.3 as well as 9q33–q34, 1q21–q25/1p11–p32 and 19p13.1–p13.4 are paralogous regions [33] that share members of the same gene family. Further analysis has shown that paralogues of human MHC genes are also scattered essentially over all chromosomes [18]. Likewise, genome-wide duplications have recently been examined in zebrafish [31], confirming that an extra round of a whole-genome duplication event occurred early in the teleost lineage after it split from the tetrapod lineage. Evidence that paralogous genes exist in zebrafish became apparent when many duplicates associated with the MHC, and mainly the class III region, were identified (Figure 1). Here we discuss five MHC-encoded gene families in more detail.

The Notch gene family members encode evolutionary transmembrane receptors that regulate cell fate determination. Four Notch paralogues (*NOTCH1-4*) have been identified in human, and only three of their orthologues are found in zebrafish (Table 3). The orthologue of human *NOTCH4*, found in the MHC, is absent in the fish whole-genome assembly, indicative that gene loss has followed the block duplication events in teleosts. This is also seen in Fugu [24] and may be due to it being the most divergent member of the Notch family [34]. Two duplicates of *notch3 *are found in scaffolds 1523 and 285. Until the assembly is complete, it is not clear whether there are two copies of this gene or if this is due to the status of the current assembly. Only one surrounding *rdh8-like *sequence is found in common between the two scaffolds. In addition, *notch1 *and *notch2 *have been mapped to chromosome 5 and contig NA15389.

Retinoid receptors are soluble nuclear proteins belonging to the steroid/thyroid hormone receptor superfamily of transcriptional regulators. The *RXR *subfamily consists of three polypeptide chains, namely alpha, beta and gamma, encoded by separate loci. The human *RXRB *gene is found within the MHC, while the *RXRA *and *RXRG *paralogues are located on chromosomes 9 and 1, respectively. Five *rxr *loci have been identified in the zebrafish assembly in contig NA16779 and chromosomes 5, 9, 17 and 21. Two semi-orthologues of human *rxrb *appear to have arisen from a fish-specific duplication, and duplicates of *rxra *are also present in the zebrafish genome. Mapping data from the current assembly in comparison to the ZFIN linkage maps are contradictory and may highlight problems in both approaches to mapping genes. Interestingly, neither of the *rxrb *sequences map to chromosome 19 in the Zv4 whole-genome assembly as originally thought [35].

The human *PBX2 *gene encodes a homeodomain-containing protein. Three paralogues are located within chromosomes 1q23, 9q33 and 19p12, named *PBX1*, *PBX3 *and *PBX4*, respectively. Four *PBX-like *sequences were identified in the zebrafish: *pbx1a *on scaffold NA14559 and chromosome 11; *pbx3b *on chromosome 18; and *pbxy *on scaffold NA11844. These are related to human *PBX1*, *PBX3 *and *PBX4*, respectively [36]. The orthologous sequence to human *PBX2*, which is present in the MHC, has not been identified in the current assembly.

The complement component *C4 *gene encodes a protein that plays a central role in the innate immune response. Structurally, C4, C5 and C3 belong to the α2 macroglobulin (A2M) protein family and are derived from a single common ancestor. In human, they are found on four different chromosomes: 6p21 (*C4*), 9q33 (*C5*), 19p13 (*C3*) and 12p13 (*A2M*), and a similar situation exists in all tetrapods studied to date. In zebrafish, the *a2m *and *c4 *genes are both found on chromosome 15 as previously shown [37]. Multiple copies of *a2m *have also been observed in zebrafish with a duplicate being present in NA17328. Two copies of the *c3 *gene were identified on chromosome 22 and NA14479.

Six members of the chloride intracellular channel (*CLIC*) gene family (*CLIC1–CLIC6*) have been described in humans. They are involved in chloride ion transport within various subcellular compartments [38]. Four *clic *genes were identified in zebrafish on chromosomes 14, 16, 17 and 18. On phylogenetic analysis, they cluster with mammalian *CLIC2*, *CLIC3*, *CLIC4 *and *CLIC5 *genes, respectively. The orthologue of the human MHC-embedded *CLIC1 *was not found.

## Conclusion

Comparative genomics reveals that the organisation of the MHC in more distantly-related organisms varies from the human model [39]. Teleost fish are particularly unusual in their organisation in comparison to mammals, chicken, Xenopus and shark in that their class I and class II loci are found on different chromosomes [14]. The core class I region in zebrafish, medaka, Fugu and rainbow trout [13,40-42] comprises genes involved in class I peptide presentation and processing: the classical class I molecules, the immunoproteasome subunits, ATP-binding cassette transporters and tapasin. The whole-genome shotgun data for zebrafish has allowed a genome-wide analysis of major histocompatibility genes and their paralogues, and highlights that one or more copies exist of MHC-related genes in fish as in humans. The results obtained thus far extend the linkage information regarding major histocompatibility genes on chromosome 19 in zebrafish and the classical mammalian MHC, and further supports previous findings that the functional class II loci are found on a different chromosome. The distribution of the remaining MHC-related genes and their paralogues is genome-wide, confirming a fragmented MHC in fish.

## Methods

The genome of the zebrafish is currently being sequenced at the Sanger Institute [43]. The analysis was carried out on the fourth Ensembl whole-genome assembly Zv4, released on September 2004 [32]. This assembly integrates the whole-genome shotgun assembly with data from the physical map [44]. Protein sequences encoded in 117 human MHC genes were chosen for BLAST sequence similarity searches against the zebrafish Ensembl assembly. Previously identified MHC-related zebrafish cDNAs [45] were also used in the analysis. Gene annotations were verified using VEGA [46]. A number of potentially duplicated genes were identified. Their predicted amino acid sequences were then aligned with other vertebrate gene family members present in the UniProt database [47] using CLUSTAL [48], and the multiple sequence alignments were used for phylogenetic analysis by the neighbour joining method [49] via the PHYLO_WIN interface [50]. Official gene symbols assigned by the Zebrafish Nomenclature Committee (ZNC) and HUGO Nomenclature Committee (HGNC) have been used in the annotation. Zebrafish and human gene names are given in lower and upper case, respectively.

The zebrafish source DNA for the whole-genome assembly was acquired from approximately 1000 five day old embryos. This has resulted in possible misassemblies due to polymorphisms between sequences, different haplotypes, and difficulties with the assembly of duplicated genes and regions, in particular with many highly homologous MHC genes that have arisen by duplication. Where possible, finished clones [e.g. EMBL:AL672216, EMBL:AL672151, EMBL:AL672185, EMBL:AL672164, EMBL:AL672176] were used to study the short-range linkage of genes in preference to the Ensembl whole-genome assembly. Alternative mapping information, for comparison and validity of the *in-silico *approach used during this study, was obtained from the Zebrafish Information Network (ZFIN) [45] and published scientific literature.

## Authors' contributions

JS carried out the analysis on the whole-genome assembly and drafted the manuscript. FF contributed materials and data. SB conceived the study and contributed towards the preparation of the manuscript.

## Note added in proof

At least one of the genes stated to be missing in assembly Zv4 is present in assembly Zv5 which was released on October 2005.
